# Queuosine Biosynthesis Is Required for *Sinorhizobium meliloti-*Induced Cytoskeletal Modifications on HeLa Cells and Symbiosis with *Medicago truncatula*

**DOI:** 10.1371/journal.pone.0056043

**Published:** 2013-02-08

**Authors:** Marta Marchetti, Delphine Capela, Renaud Poincloux, Nacer Benmeradi, Marie-Christine Auriac, Aurélie Le Ru, Isabelle Maridonneau-Parini, Jacques Batut, Catherine Masson-Boivin

**Affiliations:** 1 INRA, Laboratoire des Interactions Plantes-Microorganismes (LIPM), UMR441, Castanet-Tolosan, France; 2 CNRS, Laboratoire des Interactions Plantes-Microorganismes (LIPM), UMR2594, Castanet-Tolosan, France; 3 CNRS-IPBS (Institut de Pharmacologie et de Biologie Structurale), Toulouse, France; 4 Université de Toulouse, UPS (Université Paul Sabatier), IPBS, Toulouse, France; 5 Institut de Biologie Cellulaire et de Génétique IBCG CNRS, Toulouse, France; 6 Plateforme de Microscopie FRBT - Centre de Biologie du Développement, Toulouse, France; Centre National de la Recherche Scientifique, Aix-Marseille Université, France

## Abstract

Rhizobia are symbiotic soil bacteria able to intracellularly colonize legume nodule cells and form nitrogen-fixing symbiosomes therein. How the plant cell cytoskeleton reorganizes in response to rhizobium colonization has remained poorly understood especially because of the lack of an *in vitro* infection assay. Here, we report on the use of the heterologous HeLa cell model to experimentally tackle this question. We observed that the model rhizobium *Sinorhizobium meliloti*, and other rhizobia as well, were able to trigger a major reorganization of actin cytoskeleton of cultured HeLa cells *in vitro*. Cell deformation was associated with an inhibition of the three major small RhoGTPases Cdc42, RhoA and Rac1. Bacterial entry, cytoskeleton rearrangements and modulation of RhoGTPase activity required an intact *S. meliloti* biosynthetic pathway for queuosine, a hypermodifed nucleoside regulating protein translation through tRNA, and possibly mRNA, modification. We showed that an intact bacterial queuosine biosynthetic pathway was also required for effective nitrogen-fixing symbiosis of *S. meliloti* with its host plant *Medicago truncatula*, thus indicating that one or several key symbiotic functions of *S. meliloti* are under queuosine control. We discuss whether the symbiotic defect of *que* mutants may originate, at least in part, from an altered capacity to modify plant cell actin cytoskeleton.

## Introduction

Whereas intracellular infection by bacteria is widespread in the animal kingdom, most often as part of a pathogenic interaction, it is more sporadic in the plant kingdom. One noticeable instance is the intracellular colonization of legume root nodules by phylogenetically diverse bacteria collectively called rhizobia [1]. Rhizobia enter a mutualistic symbiosis with legumes resulting in a chronic infection of legume cells by endosymbiotic bacteria that fix nitrogen to the benefit of the plant. The plant, in turn, provides hosted bacteria with carbon resources and a privileged niche.

Rhizobia vary in their mode of initial penetration of root tissues [2]. Some enter root tissues by crack entry at the emergence of lateral roots whereas rhizobia that nodulate temperate legume such as alfalfa, soybean or clover use a more sophisticated strategy based on the formation of specialized infection structures called infection threads. Infection threads are initiated at the tip of root hairs, extend therein and propagate in the root cortex and developing nodule tissues underneath [3]. Bacteria are released at the extremity of infection threads inside the differentiating nodule cells and become enclosed in a plant-derived peribacteroid membrane to form symbiosomes, reminiscent of phagosomes found in animal systems. One single nodule cell typically contains thousands of symbiosomes.

Key molecules for nodulation and infection thread formation are the well-described lipo-chitooligosaccharides called Nod factors that are specifically recognized by plant receptor-like kinases [3], [4]. Bacterial surface lipo- and exopolysaccharides, such as low molecular weight succinoglycan, are also often required for successful infection thread formation although their actual role has not been completely elucidated yet [3]. The process of intracellular infection that was recently shown to involve both exocytic and endocytic cellular pathways [5], remains poorly understood because whole plant assays do not permit specific insight into this late symbiotic infection step.

In a search for a suitable experimental assay, we reasoned that symbiosome formation by rhizobia was reminiscent of the chronic invasion of animal cells by intracellular pathogens such as *Brucella*, *Salmonella* or *Legionella* (reviewed in [6]). Some of these animal pathogens are phylogenetically interspersed with rhizobia (*e.g. Brucella* or *Bartonella* and *Sinorhizobium*) and common genetic determinants of symbiosis and pathogenicity have been reported [6]–[8]. This prompted us to assay and co-culture *in vitro* the model rhizobium *Sinorhizobium meliloti* [9] with human HeLa cells, a widely-used experimental infection model for animal pathogens for which many molecular and cellular tools are available.

Here we report that *S. meliloti*, and other phylogenetically distant rhizobia as well, were able to induce major actin cytoskeleton rearrangements on HeLa cells. We isolated mutants of the *S. meliloti* wild-type strain that were defective for actin cytoskeleton modifications on HeLa cells. We found that these mutants were affected in queuosine biosynthesis, a modified nucleoside that affects gene expression post-transcriptionally. We showed that an intact queuosine biosynthetic pathway was also required for efficient symbiosis with *Medicago truncatula*. Possible links between actin cytoskeleton modification, queuosine biosynthesis and symbiotic proficiency are discussed.

## Results

### 1. Rhizobia induce drastic actin cytoskeleton modifications on HeLa cells

**Figure 1:**
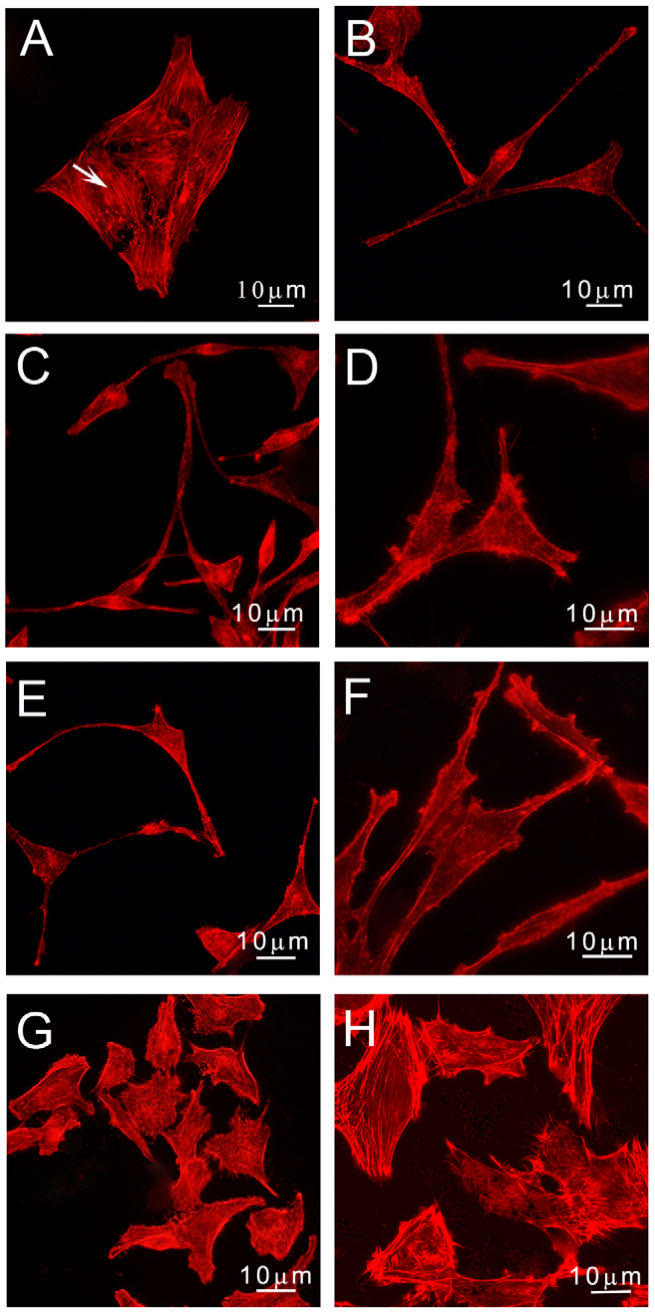
Bacteria-induced cytoskeleton modifications of HeLa cells. HeLa cells untreated (A), inoculated with *S. meliloti* (B), *R. leguminosarum* (C), *A. caulinodans* (D), *C. taiwanensis* (E), *B. tuberum* (F), *C. crescentus* (G) and *E. coli* (H). HeLa cells were stained with phalloidin-Texas red and observed by fluorescence microscopy 48 hours after bacterial inoculation. Arrow: stress fiber.

Subconfluent HeLa cells were incubated with *S. meliloti* bacteria at a multiplicity of infection of 100 (*i. e.* 100 bacteria per eukaryotic cell). The morphology of HeLa cells was observed at different time points after bacterial inoculation and fluorescent phalloidin staining of actin. Epithelial cells infected with the wild type strain 1021 of *S. meliloti* displayed an extended, slender and elongated morphology that was not observed in non-infected cells (Fig. 1AB and Fig. S1). Cell deformations could be observed *ca* 30 hours after inoculation (hpi) and increased with time so that 80% of Hela cells presented drastic morphological changes at 48 hpi. *S. meliloti* also induced a loss of stress fibers in HeLa cells (Fig. 1AB) as well as a block in cell cycle (Fig. S2).

Rhizobia are phylogenetically disparate bacteria among the α- and β-subclasses of proteobacteria that share the ability to form an endocellular symbiosis with plants [1]. We thus tested rhizobia belonging to different genera for their impact on HeLa cell morphology. *Rhizobium leguminosarum*, *Azorhizobium caulinodans* and the β-rhizobia *Cupriavidus taiwanensis* and *Burkholderia tuberum* induced cytoskeleton changes on HeLa cells similar to those induced by *S. meliloti* (Fig. 1). Instead, no cytoskeleton modifications were observed upon inoculation of HeLa cells with the aquatic bacterium *Caulobacter crescentus* -an α-proteobacterium closely related to *S. meliloti*- and with the γ-proteobacterium *E. coli* (Fig. 1, Fig. S3). This set of observations indicated that different rhizobia share the ability to promote actin cytoskeletal rearrangements on eukaryotic cells, which could be symbiotically relevant [10], [11].

This result prompted us to explore in more detail the molecular mechanisms underlying the cellular changes induced by *S. meliloti* strain 1021 on HeLa cells. No cellular modification was observed when HeLa cells were separated from *S. meliloti* cells by a 0.2 µm anapore membrane or incubated with a bacteria-free culture supernatant (data not shown). This suggested that cytoskeleton modifications were not provoked by a diffusible bacterial molecule and, instead, required a physical contact between bacteria and HeLa cells. Heat-killed *S. meliloti* cells did not trigger morphological changes thus indicating that live bacteria were needed for HeLa cell deformation (data not shown).

**Figure 2:**
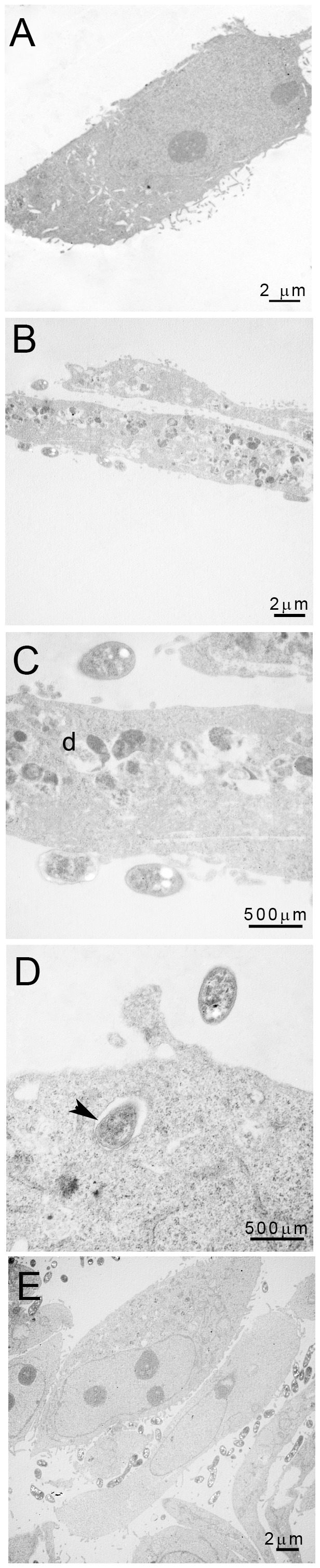
Intracellular infection of HeLa cells by *S. meliloti*. HeLa cells were observed by electron microscopy 48 hpi (A): non-inoculated control (B–D): HeLa cells inoculated with wild-type *S. meliloti* 1021 (E): Hela cells inoculated with a *queF* mutant. d: degraded bacterium. Arrowhead: internalized bacterium.

HeLa cells were inoculated with a GFP-tagged *S. meliloti* strain in order to monitor bacterial entry and survival. Confocal analyses showed rare (2–3/cell) live, GFP-expressing, bacteria in *ca* 50% of HeLa cells, between 18 and 48 hpi (Fig. S4). Instead, electron microscopy analyses showed a high number of intracellular bacteria within vacuoles, most often in a degraded state thus indicating that bacteria were efficiently internalized but did not maintain (Fig. 2).

**Figure 3:**
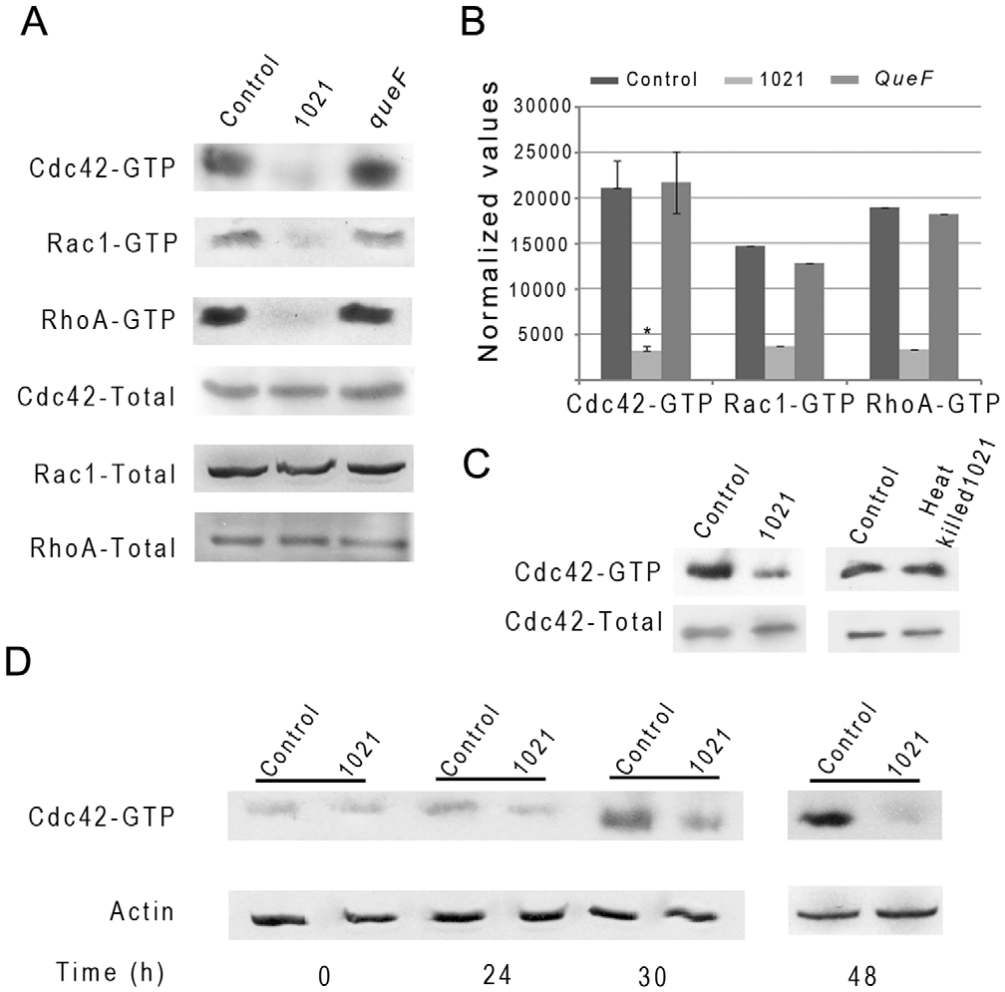
Determination of GTPases activation state in bacteria-treated HeLa cells. (A) Representative pull down assays of active Cdc42, Rac1 and RhoA GTPases at 48 hpi in non-inoculated- (control), *S. meliloti* 1021- and *queF*-inoculated HeLa cells. (B) Quantification of pull down assays using ImageJ software. Means ± S.D. were calculated from three independent experiments for Cdc42-GTP and mean from two independent experiments for Rac1-GTP and RhoA-GTP. Results were normalized to the corresponding total protein. Statistical significance (P<0.001) is shown (*) with respect to the control. (C) Immunoprecipitation of active and total CdC42 from non-inoculated (control) HeLa cells or cells inoculated with live and heat-killed wild-type bacteria 48 hpi. (D). Kinetics of Cdc42 activation. Actin, total Cdc42, Rac1 and RhoA or active GTP-bound forms of Cdc42, Rac1 or RhoA were detected by immuno-blotting of SDS-PAGE gels.

As small GTPases Cdc42, Rac1 and RhoA are known to coordinate signaling cascades that produce both morphological and nuclear responses to a variety of extracellular signals [12], [13] we examined whether these GTPases were involved in the *S. meliloti*-induced cellular responses of HeLa cells. The activation state of Cdc42, Rac1 and RhoA of HeLa cells challenged with *S. meliloti* was measured by pull down assays (Fig. 3). At different time points following HeLa cell infection with *S. meliloti*, lysates were prepared and the amount of active, GTP-bound, Cdc42, Rac1 and RhoA precipitated with the GST-CRIB fusion protein was determined by western blotting [14]. A decrease in the level of the active form of the three small RhoGTPases was detected at 48 hpi with live *S. meliloti* (Fig. 3AB) that was not detected after heat-killing of bacteria (Fig. 3C). A kinetic analysis showed that Cdc42 inhibition was already detectable at 24 hpi (Fig. 3D).

### 2. HeLa cell invasion, cytoskeleton modifications and modulation of RhoGTPases activity by *S. meliloti* required an intact queuosine biosynthetic pathway

**Figure 4:**
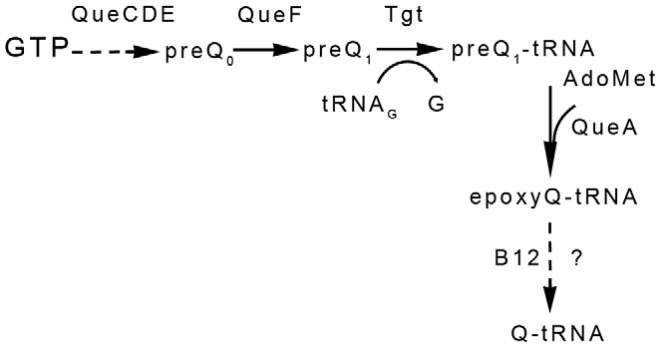
The *S. meliloti* queuosine biosynthetic pathway. preQ_0_: 7-cyano-7-deazaguanine, preQ1: 7-(aminomethyl)-7-deazaguanine, AdoMet: S-adenosyl-L-methionine, EpoxyQ: epoxyqueuosine, Q: queuosine. Adapted from [49], [50].

With the aim to identify bacterial genes involved in HeLa cell infection and cytoskeleton modifications, we screened a library of *S. meliloti* mutants for their ability to induce HeLa cell elongation. Mutants in genes known to be essential for symbiosis such as genes involved in Nod Factor production (*nodA*, *nodD1nodD2nodD3*), exopolysaccharide (*exoITWY*) or lipopolysaccharide (*lpsB*) synthesis, bacteroid differentiation and survival (*bacA*), nitrogen fixation and microoxic respiration (*fixJ*), stress adaptation (*typA*) or the crp-like regulator Clr (Table S1) all triggered HeLa cells deformations as wild-type thus indicating that cytoskeleton modifications were independent from known symbiotic genes. Upon testing *S. meliloti* strains carrying large deletions on the pSymB megaplasmid [15], we found that a 120 Kb-large deletion (Rm541) prevented HeLa cell elongation. Nested site-directed deletions in the same region (GMI11660, GMI11661, GMI11662; Table S1) indicated that the locus responsible for HeLa cell deformation mapped into the *exs* gene cluster required for succinoglycan biosynthesis [16]. Systematic individual inactivation of genes located in this region showed that the *exsBCD* gene cluster was responsible for actin cytoskeleton modifications on HeLa cells. Expert sequence analysis of the *exsBCD* genes indicated that they are the likely orthologues of the *queCDE* genes from *E. coli* and *B. subtilis* involved in the biosynthesis of queuosine (Fig. 4), a modified nucleoside that replaces guanosine at position 54 of tRNAs_GUN_ [17]. We thus renamed the *exsBCD* genes of *S. meliloti queCDE*.

**Figure 5:**
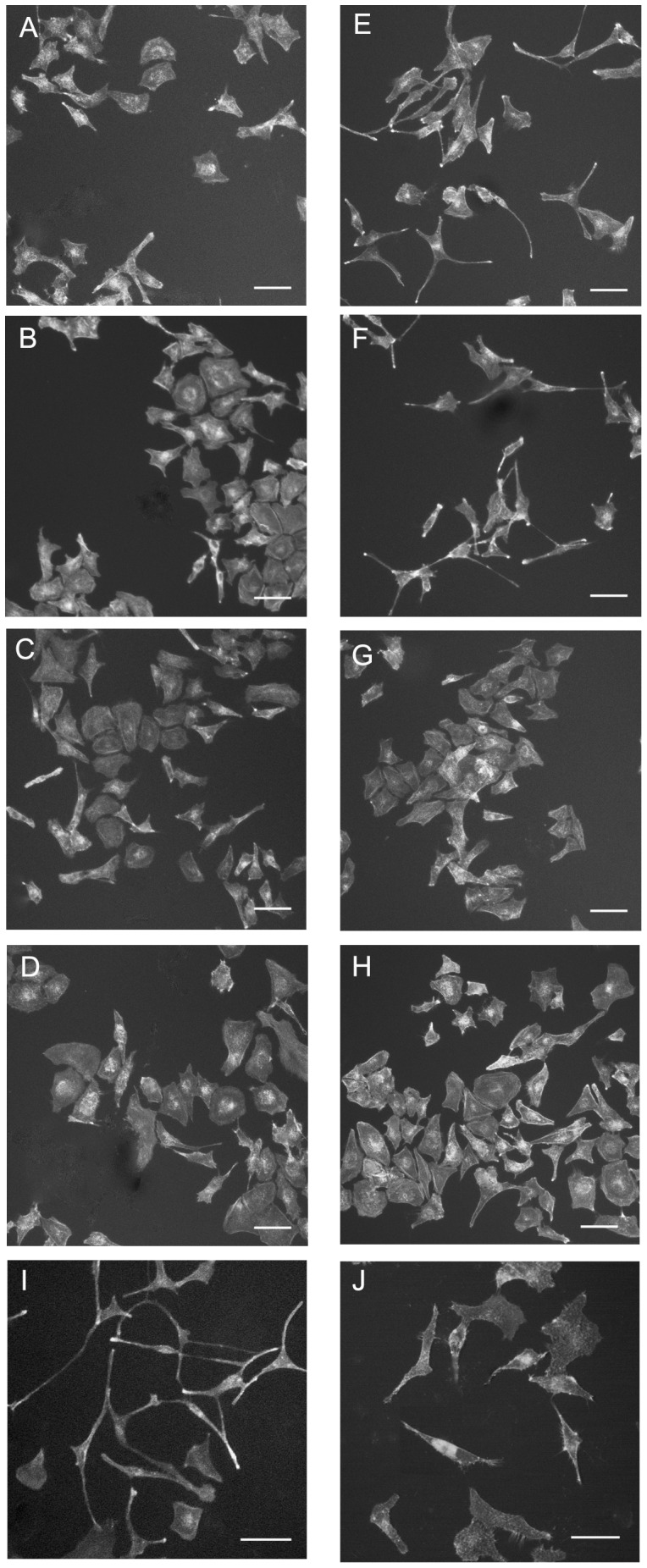
Cytoskeleton modifications of HeLa cells infected with *S. meliloti* queuosine biosynthesis mutants. HeLa cells were incubated with *S. meliloti queC* (A, E), *queF* (B, F), *tgt* (C, G), *queA* (D, H), the wild type 1021 strain (I) and the *queF* complemented strain (GMI11186) (J) in 0.5% FCS culture medium alone (A–D, I, J) or supplemented with preQ1 (E–H). HeLa cells were stained with phalloidin-Texas red and observed by fluorescence microscopy 48 hpi. Scale bar: 10 µm.

Looking for additional genes belonging to this pathway (Fig. 4) we identified *queF* (SMc02723), *tgt* (SMc01206) and *queA* (SMc01207) genes on the *S. meliloti* main chromosome. These genes were individually inactivated and corresponding mutants triggered reduced HeLa cell deformations 48 hpi in regular HeLa culture medium (DMEM) containing 10% Foetal Calf Serum (FCS). As FCS is known to contain queuine [18], the base moiety of queuosine, we cultured HeLa cells in a medium containing only 0.5% FCS. We then observed that the *queC*, *queF* and *tgt* mutants were completely unable to induce actin cytoskeletal changes on HeLa cells in this medium (Fig. 5). This was not due to an altered growth of *que* mutants as compared to wild-type under the assay conditions (Fig. S5). Supplementation of the 0.5% FCS culture medium with 300 nM of the queuosine precursor preQ1 completely restored an elongated HeLa phenotype for the *queC* and *queF* mutants, but not for the *tgt* or *queA* mutant consistently with the proposed queuosine biosynthetic pathway (Fig. 4). Genetic complementation of the *queF* null mutant by a plasmid-encoded *queF* gene (GMI11686, Table 1) restored HeLa cell deformation, as expected (Fig. 5J). Pull down experiments performed on HeLa cells inoculated with the *queF* mutant at 48 hpi revealed high activity of the Rho-GTPases, as in non-inoculated cells or cells inoculated with heat-killed bacteria (Fig. 3AB), thus reinforcing the link between Rho-GTPases inhibition and actin cytoskeleton modifications.

**Table 1:** Bacterial strains and plasmids.

Strains	Description^a^	Reference
*S. meliloti strains*		
1021	Str^R^ derivative of *S. meliloti* strain SU47	[51]
Rm5416	ΔΩ5007-5011::Tn5-233, Gen^R^, Spe^R^	[52]
GMI11655	1021 queA::pVO155 (221 bp), Str^R^, Neo^R^	This work
GMI11656	1021 queC::pVO155 (63 bp), Str^R^, Neo^R^	This work
GMI11657	1021 tgt::pVO155 (128 bp), Str^R^, Neo^R^	This work
GMI1546	1021 queF:pVO155 (18 bp), Str^R^, Neo^R^	This work
GMI11658	1021 SMc02721::pVO155 (654 bp), Str^R^, Neo^R^	This work
GMI11659	1021 SMc02722::pVO155 (881 bp), Str^R^, Neo^R^	This work
GMI11669	1021 pHC60 Str^R^ Tet^R^	This work
GMI11686	GMI11546 pGMI(queF), Str^R^, Neo^R^, Gen^R^	This work
*Other bacterial strains*		
248	*Rhizobium leguminosarum bv viciae*	[43]
ORS571	*Azorhizobium caulinodans*	[44]
LMG19424	*Cupriavidus taiwanensis*	[45]
STM678	*Burkholderia tuberum*	[46]
NA1000	*Caulobacter crescentus*	[47]
DH5 alpha	*Escherichia coli*	[42]
*Plasmids*		
pHC60	pHC41 containing GFP-S65T, Tet^R^	[53]
pRK600	Helper plasmid, Chl^R^	[52]
pVO155	Suicide plasmid, Kan^R^, Amp^R^	[41]

a The location of plasmid insertions (number of nucleotides after the start codon) is indicated between brackets.

Electron microscopy indicated that HeLa cells were not, or very poorly, invaded by the *queF* mutant (Fig. 2E). We thus wondered whether the defect of this mutant in promoting actin cytoskeleton reorganization originated from its defect in invading HeLa cells. This was ruled out by treating HeLa cells with the *E. coli* CNF-1 toxin, that is known to promote internalization of bacteria in cultured cells [19]. CNF-1 restored entry of the *queF* mutant without restoring actin cytoskeleton modifications (Fig. S4). Furthermore, the CNF-1 toxin promoted an increase in wild-type bacteria internalization, as compared to untreated cells, without exacerbating or accelerating the extent of cytoskeleton modifications (data not shown). This suggests that bacterial entry might not be required for cytoskeleton modifications.

### 3. A *S. meliloti* 1021 *queF* mutant is severely affected in late symbiotic stages

**Figure 6:**
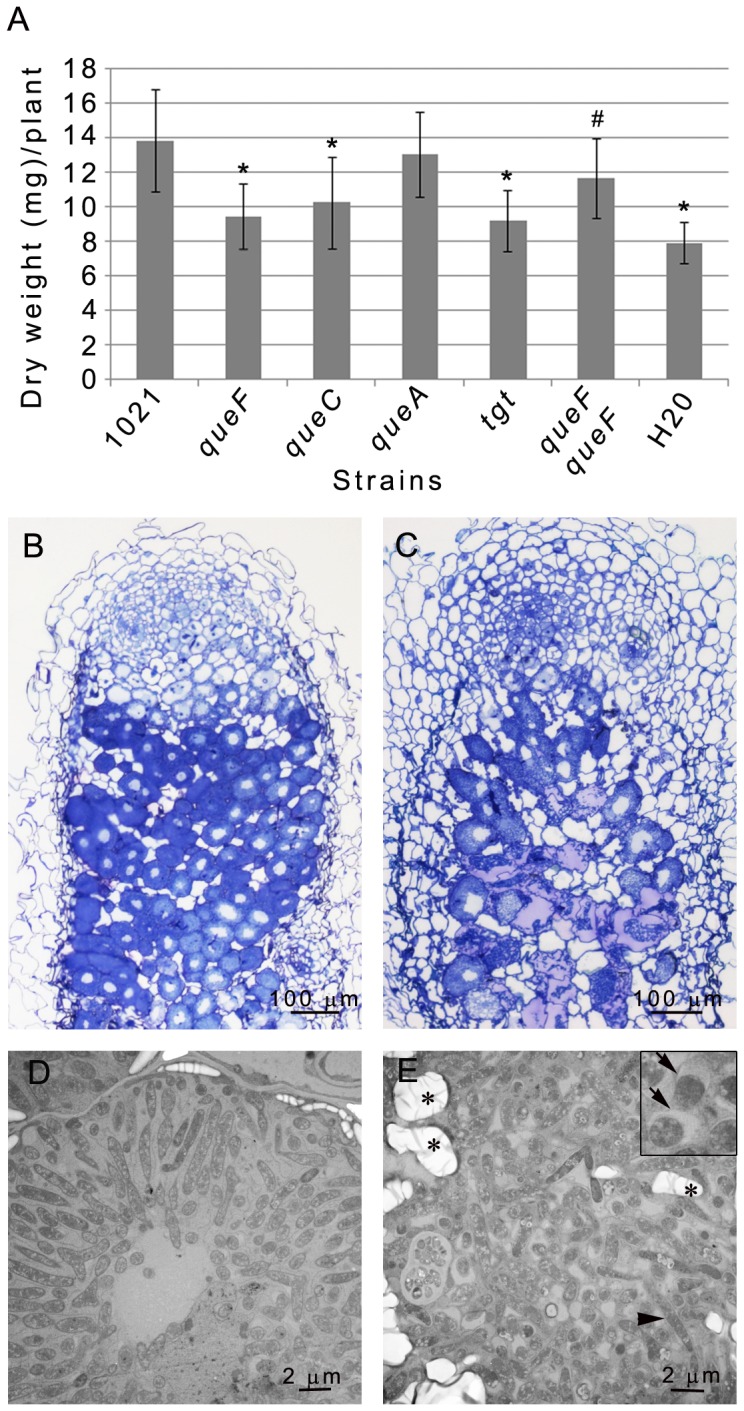
Symbiotic phenotype of *S. meliloti* queuosine mutants. Dry weight of *M. truncatula* seedlings inoculated with *S. meliloti* 1021, different queuosine-deficient mutants and the *queF* complemented (GMI11186) strain at 40 dpi. Statistical significance (P<0.01) is shown with respect to strain 1021(*) and the *queF* mutant (^#^), respectively. (B, C) Sections of *M. truncatula* 21–day old nodules induced by 1021 (B) and the *queF* isogenic mutant (C). (D, E) Electron micrographs of nodule cells infected with 1021 (D) or the *queF* mutant (E). *queF* mutant bacteroids are randomly organized within the infected cell whereas 1021 bacteroids show a radial organization. (Insert panel in E): arrows point to symbiosome membranes detached from *queF* bacteria (). Arrowhead, type 4/5 bacteroid. *, starch granules.

We assessed the symbiotic performances of the various *S. meliloti* queuosine deficient mutants on the model legume *Medicago truncatula*. At 40 dpi, aerial parts of *Medicago* plants grown in nitrogen-free medium and inoculated with the mutants were small and yellowish suggesting a defect in nitrogen fixation. Dry-weight measurements of aerial parts confirmed a profound defect of the *queF*, *queC and tgt* mutants in nitrogen fixation ability (Fig. 6A). Surprisingly, no or little symbiotic phenotype was observed for the *queA* mutant. Genetic complementation of the *queF* null mutant by a wild type allele restored symbiotic proficiency (Fig. 6).

A more detailed phenotypic characterization of plants inoculated with the *queF* mutant was performed. The kinetics of nodulation by the *queF* mutant was indistinguishable from wild-type (Fig. S6), thus pointing to a late symbiotic defect. The indeterminate nodules formed by *S. meliloti* on *M. truncatula* display a longitudinal gradient of bacteroids at different stages of differentiation, the most differentiated forms (type 4/5 bacteroids) in the central zone of the nodule being responsible for nitrogen fixation [20]. Optical and electron microscopy analyses of *queF*-induced nodules showed a sharp decrease in the number of successfully infected cells (Fig. 6C). Bacteroids formed by the *queF* mutant differentiated into type 4/5 bacteroids (Fig. 6E) that were, however, randomly organized within the infected cells, in striking contrast to the normal radial organization of wild-type *S. meliloti* bacteroids (Fig. 6D). This indicates a defect in symbiosome organization in *queF*-infected nodules. Numerous starch granules were observed consistent with the carbon imbalance resulting from the defect in nitrogen fixation. Finally, an early degeneration of infected nodule cells was observed as soon as 21 dpi. At 40 dpi, no more live *queF* bacteria were observed in nodule cells indicating a strong defect in bacteroid maintenance (data not shown).

## Discussion

Despite obvious intrinsic limitations, *in vitro* assays and surrogate hosts are useful simplified experimental systems to help deciphering complex biological processes taking place during host-microbe interactions [21]. Our understanding of rhizobial intracellular infection, a late step in the symbiotic interaction, has been so far impaired by the lack of a dedicated assay. In this study, we report on the ability of *S. meliloti* to trigger a dramatic rearrangement of actin cytoskeleton on HeLa cells, resulting in severely elongated cells, reminiscent of the “hummingbird” phenotype induced by *Helicobacter pylori* [22]. As with *H. pylori*, a long (48 hpi) incubation time was needed to observe a full cytopathic effect on HeLa cells [23]. Interestingly, other phylogenetically distant rhizobia induced similar modifications on HeLa cells, whereas *C. crescentus* and *E. coli* did not.

Actin cytoskeleton rearrangements are known to occur at different stages of the symbiotic interaction between rhizobia and their host plants, including root hair growth and curling, infection thread formation, bacterial internalization and positioning in plant nodule cells, cell trafficking and possibly defence reactions [24], [25]. It is thus tempting to speculate that the actin cytoskeleton deformations elicited by rhizobia on HeLa cells may reflect the ability of these bacteria to manipulate legume plant actin cytoskeleton. In animal systems, actin-based cytoskeleton rearrangements are regulated by small GTPases of the Rho family, which are often hijacked by animal pathogens [26], [27]. We took advantage of the molecular tools available on human Rho-GTPases to demonstrate that the actin cytoskeleton modifications induced by *S. meliloti* on HeLa cells were associated with an inhibition of the activity of the three major Rho-GTPases, RhoA, Rac and Cdc42. This suggests that *S. meliloti* and possibly other rhizobia may manipulate plant Rho-GTPases in a symbiotic context.

Further experiments are now required to assess this possibility in a homologous context. In contrast to animal and fungi, plants possess a single family of closely related members known as ROPs (Rho-GTPases of plants), similar to animal Rac-GTPases [28]. *Medicago and Lotus* Rho-GTPases-encoding genes have been identified recently that express at different stages of the symbiotic interaction [28]–[32]. It would be very interesting to characterize both the expression and activity of these *Medicago* Rho-GTPases in response to infection by wt *S. meliloti* and *que* mutants.

A novel finding reported here is that actin cytoskeletal modifications as well as Rho-GTPases inhibition on HeLa cells required an intact pathway for queuosine biosynthesis. Queuosine is a hyper-modified guanosine whose function is not completely understood. Previous studies have shown that the queuosine modification of tRNAs_GUN_, specifying aspartic acid, asparagine, tyrosine and histidine, may affect the translation efficiency of target proteins by either modulating the interaction of tRNAs with the different degenerate codons [33] or by enhancing the binding efficiency of tRNAs to the ribosomes [34]. Interestingly, it was shown recently that some mRNAs could be directly queuosine-modified *in vitro* [35] suggesting that queuosine may modulate protein translation by targeting either tRNAs or mRNAs. Contrary to eukaryotes that obtain queuosine or queuine from their diet, bacteria can synthesize queuosine *de novo* [36]. We have identified the corresponding biosynthetic pathway in *S. meliloti* whose genes are split on three loci on two different chromosomes. Although the queuine base was previously shown to affect cell proliferation and intracellular signaling in HeLa cells [37], no cytological modifications similar to those described here have been reported, to our knowledge, upon queuine addition. Furthermore, we observed no cytoskeleton modification of HeLa cells upon addition of (wild-type) bacterial culture supernatants thus making it unlikely that free queuine or queuosine potentially secreted by wild-type rhizobia could be responsible for the cell deformations described here. Instead, a physical contact between live bacteria and HeLa cells was required for HeLa cell deformation to occur. Bacterial entry however might not be required for cytoskeleton reorganization, based on *E. coli* CNF1 toxin experiments. Altogether this suggests that a *S. meliloti* protein(s) whose synthesis is under queuosine control, or its product, might be involved in actin cytoskeletal modifications. In *S. meliloti*, as in *S. fredii*, the *queCDE*(*exsBCD*) gene cluster is physically linked to genes involved in exopolysaccharide synthesis. Furthermore, a *queC* (*exsB*) mutant of *S. meliloti* was shown to overproduce succinoglycan, a symbiotically important exopolysaccharide, thus indicating a functional link between the two pathways [16]. Succinoglycan itself is not responsible for eliciting HeLa cell deformation since all *exo* mutants tested, including *exoY* that is completely defective in succinoglycan production, induced HeLa cell deformation as wild-type. A *queA* mutant of *S. meliloti* also has modified lipopolysaccharides [38]. However a *lpsB* mutant affected in a mannosyltransferase required for LPS core biosynthesis and symbiosis with *Medicago sp.* [39], triggered HeLa cell deformation as wild-type (Table S1). Hence the *que*-dependent functions responsible for triggering actin cytoskeleton modifications remain to be identified.

The *queF*, *queC* and *tgt* mutants of *S. meliloti* 1021 had a severe defect in symbiotic phenotype on *M. truncatula*. Surprisingly, no conspicuous symbiotic defect was observed with the *queA* mutant. A possible reason could be the complementation of the *queA* mutant by a plant-derived metabolite or, alternatively, an alternate bacterial gene/pathway specifically expressed *in planta*. Noteworthy, we observed a parallel between the intensity of the symbiotic defect on plants and the intensity of symptoms on HeLa cells, the *queF* mutant being the most affected and the *queA* mutant the less affected in both cases. The *queF* mutant was not affected for the early symbiotic stages, including nodulation, root hair infection and infection thread formation and progression into the cortex. In contrast, the *queF* mutant displayed a decreased efficiency in nodule cell infection and, strikingly, a loss of radial organization of symbiosomes in infected cells which is indeed known to depend on actin reorganization [40]. We thus speculate that the symbiotic alterations observed with the *queF*/*queC* and *tgt* mutants may originate from an altered Rho-signaling and incomplete cytoskeleton reorganization taking place at the time of bacteroid establishment in nodule plant cells. However, the pleiotropic nature of *que* mutants precludes establishing at this stage a causal link between the altered capacity to trigger changes in actin-cytoskeleton and the symbiotic deficiency. Identifying more precisely the queuosine-dependent functions in *S. meliloti* is now required to challenge this possibility.

### 1. Experimental procedures

#### Bacterial strains and growth conditions

Bacterial strains and plasmids used in this study are listed in Tables 1 and S1. For genetic purposes, *S. meliloti* strains were grown at 28°C in TY (Tryptone-Yeast) medium supplemented with 6 mM of CaCl_2_. Antibiotics were used at the following concentrations: streptomycin 200 µg/ml, neomycin 100 µg/ml, tetracyclin 10 µg/ml. The 1021 *queA*, *queC*, *queF* and *tgt* mutants were obtained by site-specific insertion of the pVO155 plasmid as previously described [41]. Primers used for mutagenesis are listed in Table S2. pVO155 plasmids for mutants generation and the pHC60 plasmid for the construction of GFP expressing bacteria were introduced in *S. meliloti* by triparental mating using the pRK600 as helper plasmid. Mutants were checked by PCR. *Rhizobium leguminosarum*, *Azorhizobium caulinodans*, *Cupriavidus taiwanensis*, *Burkholderia tuberum*, *Caulobacter crescentus*, and *E. coli* were grown as previously described [42]–[47].

### 2. HeLa cell culture and infections

HeLa cells were routinely cultured in DMEM (Invitrogen) medium supplemented with 2 mM L-glutamine and 10% heat inactivated FCS (Invitrogen) at 37°C in a 5% CO_2_ incubator. For immunoprecipitation and electron microscopy experiments, cells were cultured in 10 cm petri dishes. For immunofluorescence experiments and confocal microscopic observations, 10^4^ cells were grown with either 10% or 0.5% FCS on glass coverslips in 24-well plates. All bacterial species were grown to mid-log phase and added to HeLa cells 24 hours after eukaryotic cell replication at a multiplicity of infection of 100. After infection, HeLa cells were grown as usual at 37°C in a 5% CO_2_ incubator. For long incubation times (>24 hours) the culture medium was replaced by fresh medium at 24 hpi. Cell-bacterium separation assays were performed using 0.2 µm Anopore™ membrane (Nunc). For supplementation experiments, the queuosine precursor preQ1 (300 nM final) was added just before bacterial inoculation. The *E. coli* CNF-1 toxin was used at 10^−8^ M and added to the culture medium just before bacterial infection.

### 3. Immunoprecipitation assays

Activation of endogenous Cdc42, Rac1 and Rho GTPase was measured by p21-activated kinase (PAK)-Cdc42-Rac1 (CRIB) and Rhotekin-Rho interaction binding pull-down assays as previously described [14]. In brief, at different time points after bacterial inoculation, HeLa cells were washed twice with cold PBS and lysed by scraping on ice in cold radio-immunoprecipitation assay (RIPA) buffer (50 mM Tris-HCl pH 7.4, 150 mM NaCl, 1% Triton X-100, 0.5% sodium deoxycholate, 0.1% SDS, 10 mM MgCl_2_). Lysates were cleared by centrifugation at 13,000 rpm for 15 min at 4°C, an aliquot was saved to assess total protein level and the remaining lysates were used to assess level of active GTPases. Lysates were incubated for 1 h at 4°C with *ca.* 30 µg of glutathione sepharose beads (GST) fused to the CRIB domain of PAK, WASP or Rotekin. GST beads (Amersham) were added (50% slurry) for 1 h at 4°C to precipitate GTPases. Beads were subsequently washed twice in cold washing buffer (50 mM Tris pH 7.5, 137 mM NaCl, 10 mM MgCl2, 1% Triton X-100). Equal amount of beads and total cell lysates were resolved by SDS-PAGE, transferred to nitrocellulose membrane and immuno-blotted using anti-Cdc42, Rac1 or Rho antibodies (Santa Cruz). Blots were visualized using chemioluminescence reagents (Amersham).

### 4. HeLa cells microscopic and flux cytometry examination

For fluorescence microscopy analysis, cells were washed 3 times with PBS and fixed with 3.7% Paraformaldehyde (PFA) for 30 min, free aldehyde groups were blocked with 50 mM NH_4_Cl for 15 minutes, then cells were permeabilized with 0.3% Triton X-100 for 10 min and stained with Texas red-labelled phalloidin (Molecular Probes) to visualize F-actin cytoskeleton. Cells were mounted with mounting medium (DakoCytomation) and observed with an inverted LEICA microscope (Axiophot I) or a confocal LEICA SP2 AOBS microscope.

For electron microscopy analysis, untreated or bacteria-inoculated HeLa cells were recovered by scraping of 9 cm Petri dishes and fixed in 2.5% glutaraldehyde in phosphate buffer. The fixed material was postfixed in 1% osmium tetroxide, dehydrated in an ethanol series and then embedded in Epon 812 for electron microscopy analysis. Semithin (1 µm) and ultrathin (0.1 µm) sections were taken using a Reichert Ultracut ultramicrotome. The semithin sections were collected on glass slides and stained with 1% toluidine-0.1% methylene blue in borax. The ultrathin sections were stained with uranyl acetate and lead citrate before being viewed under a Hitachi EM600 transmission electron microscope. For scanning electron microscopy, control or infected cells (48 hpi) were grown on 0.5 mm slides, fixed in glutaraldehyde (4%) in cacodylate buffer, washed, dehydrated, and metallized (1.2 V, 10 mA) before observation under a MAB Hitachi S450 microscope.

For cell cycle analyses, untreated and bacteria-inoculated HeLa cells were grown on 9 cm Petri dishes in DMEM medium with 0.5% FCS and proceeded as described [48]. Briefly, cells were recovered by scraping 48 hpi into PBS-EDTA 0.02% at 4°C. After centrifugation the cell pellet was washed in cold PBS and centrifuged again. Cells were then resuspended in Propidium Iodide/Triton X-100 staining solution (0. 1% (v/v) Triton X-100 (Sigma) in PBS, 2 mg DNAse-free RNAse A (Sigma), 0.40 ml of 500 µg/ml PI) and analysed on a cyto Facscalibur (Becton Dickinson).

### 5. Plant assays

Seeds of *Medicago truncatula* cv. Jemalong A17 were surface sterilized, germinated and grown in test tubes containing slanting nitrogen-free Fahraeus agar medium for three days at 22°C with day and night cycles of 16 and 8 hours, respectively. 20 plants were inoculated per strain with 2.10^3^ bacteria per plant. Nodulation kinetics was followed for *ca.* 40 days, and then aerial part of plants was collected, dried for 2 days at 65°C and weighted. For histological examination, nodules were fixed in glutaraldehyde (2.5% in phosphate buffer 0.1 M pH 7.4), osmium treated, dehydrated in an alcohol series, and embedded in Epon 812. Semithin nodule sections were observed after staining in 0.1% aqueous toluidine blue solution under a Zeiss Axiophot light microscope. Ultrathin sections were stained with uranyl acetate and observed under a Hitachi EM600 electron microscope.

## Supporting Information

Figure S1 **Scanning electron micrographs of untreated- (A) and** ***S. meliloti*** **1021-inoculated (B) HeLa cells 48 hpi.** Insert in panel B shows bacteria adhering to HeLa cells.
(TIF)

Figure S2 **Flow cytometry analysis of cell cycle of HeLa cells untreated (A) or inoculated with** ***S. meliloti*** **1021 (B) or the** ***queF*** **mutant strain (C) 48 hpi.** Cell cycle was arrested in phase G0/G1 with *S. meliloti* 1021. M1 and M2 mark the G0/G1 and the G2/M phases, respectively.
(TIF)

Figure S3 **Bacterial growth in HeLa cell culture medium (10%FCS).**
(PPTX)

Figure S4 **Confocal microscopy of HeLa control cells (A,B) or 48 h post-inoculation with GFP-expressing bacteria** ***S. meliloti*** **1021(pHC60) (C–F) or isogenic** ***queF*** **derivative (G–J) in the absence (A,C,D,G,H) or presence (B,E,F,I,J) of** ***E. coli*** **CNF1 toxin.** Cells were stained with phalloidin before microscopic analysis. A,B,C,E,G,I are z-stacks images. D,F,H,J are representations of one XZ-image. Scale bar 10 µm.
(TIF)

Figure S5 **Growth of** ***S. meliloti*** **wt 1021 and** ***que/tgt*** **isogenic derivatives in DMEM culture medium with 10%FCS or 0.5% FCS (A), during HeLa cell infection in DMEM medium with 0.5%FCS (B) and in Vincent-succinate medium (C).**
(PPTX)

Figure S6 **Nodulation kinetics of** ***S. meliloti*** **1021 and** ***queF*** **mutant on** ***M. truncatula*** **seedlings.** 20 plants were tested per strain.
(PPTX)

Table S1 **Effect of** ***S. meliloti*** **mutants on HeLa cells.**
(DOCX)

Table S2 **Oligonucleotides used for the construction of strains and plasmids.**
(DOC)
