# Supplementary figures and images for: Queuosine Biosynthesis Is Required for Sinorhizobium meliloti-Induced Cytoskeletal Modifications on HeLa Cells and Symbiosis with Medicago truncatula
Source: PLoS One. 2013 Feb 8;8(2):e56043. doi: 10.1371/journal.pone.0056043 (PMC3568095; doi:10.1371/journal.pone.0056043)

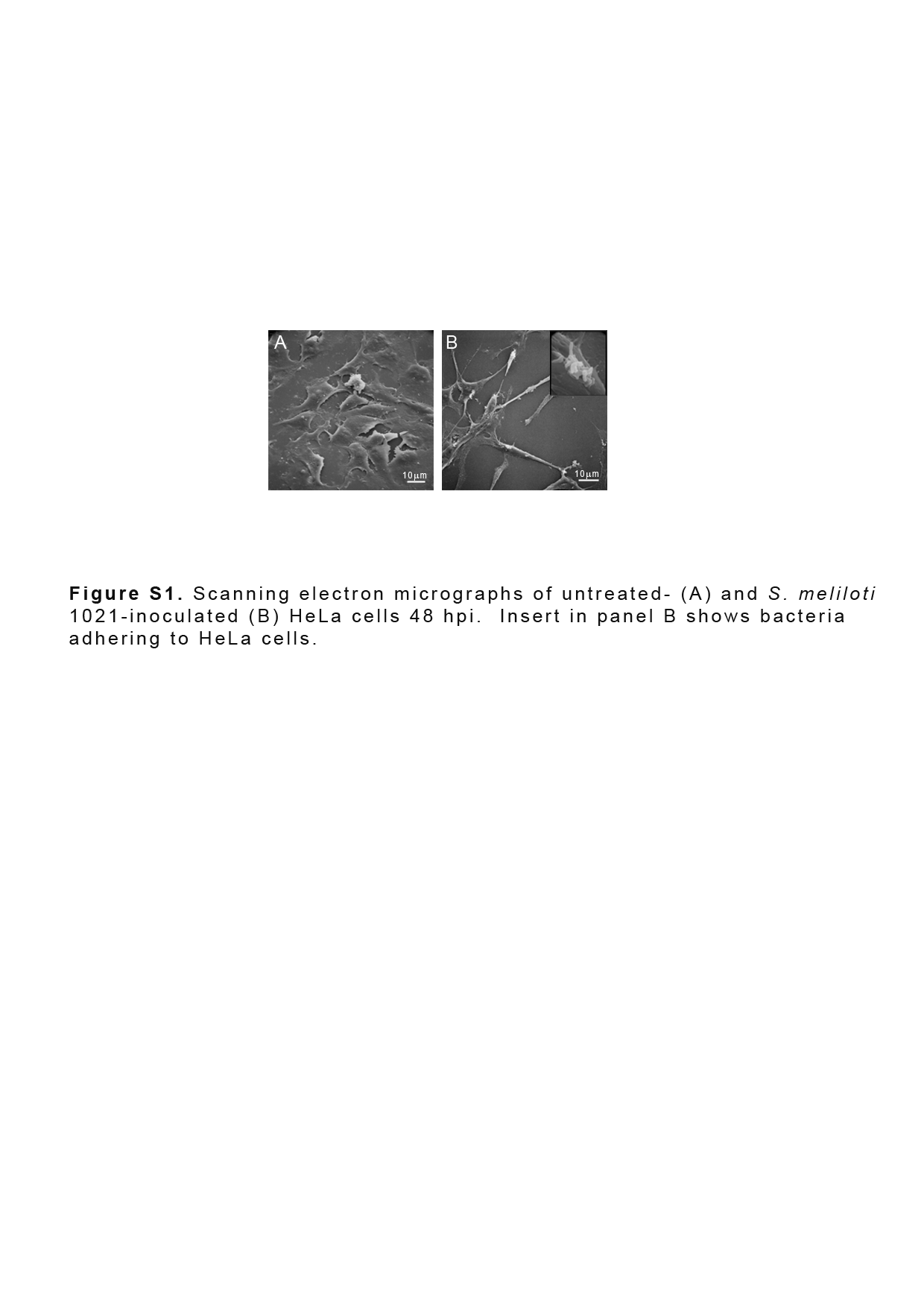

Supplement: Figure S1 — Scanning electron micrographs of untreated- (A) and S. meliloti 1021-inoculated (B) HeLa cells 48 hpi. Insert in panel B shows bacteria adhering to HeLa cells. (TIF) [file pone.0056043.s001.tif]

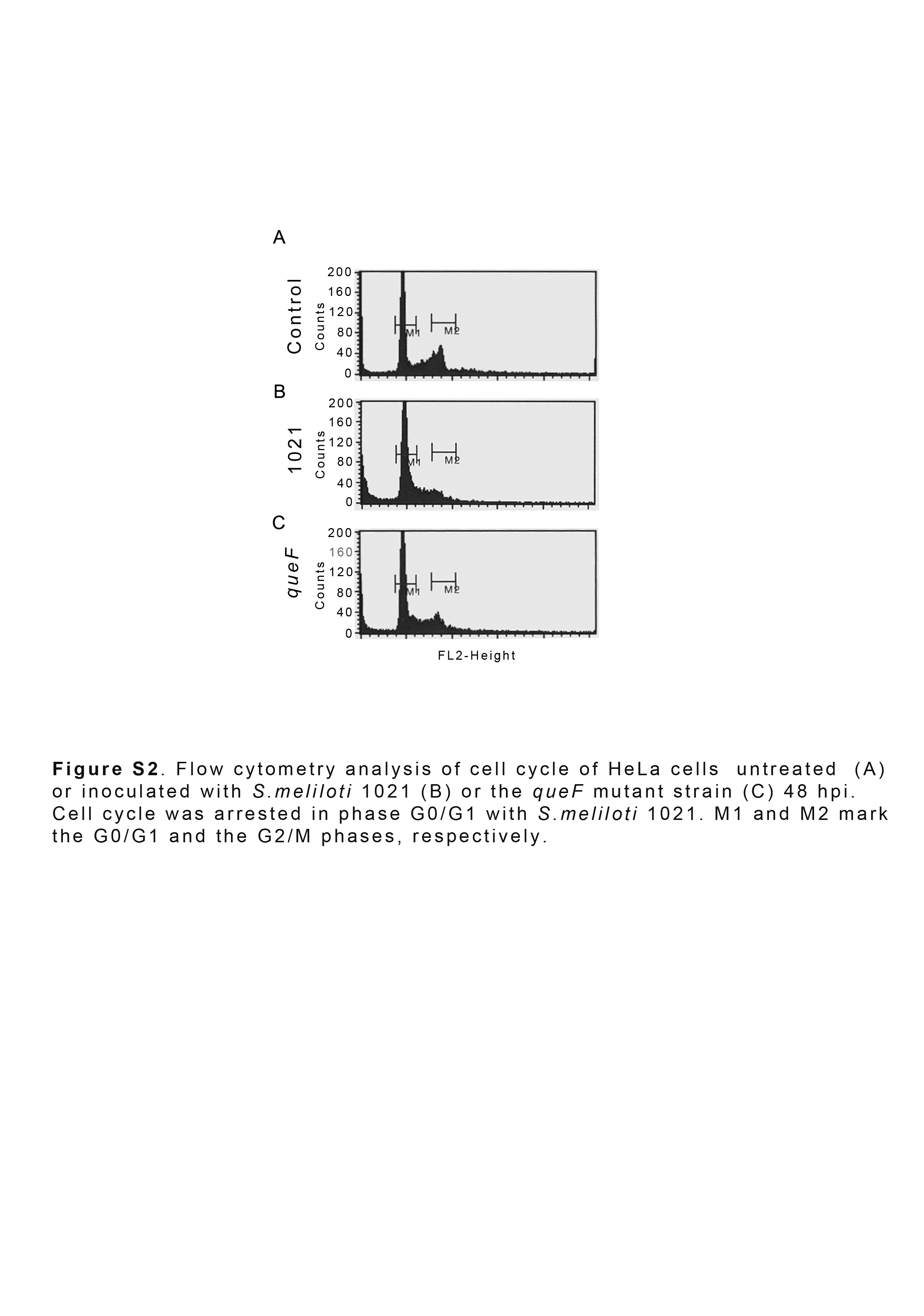

Supplement: Figure S2 — Flow cytometry analysis of cell cycle of HeLa cells untreated (A) or inoculated with S. meliloti 1021 (B) or the queF mutant strain (C) 48 hpi. Cell cycle was arrested in phase G0/G1 with S. meliloti 1021. M1 and M2 mark the G0/G1 and the G2/M phases, respectively. (TIF) [file pone.0056043.s002.tif]

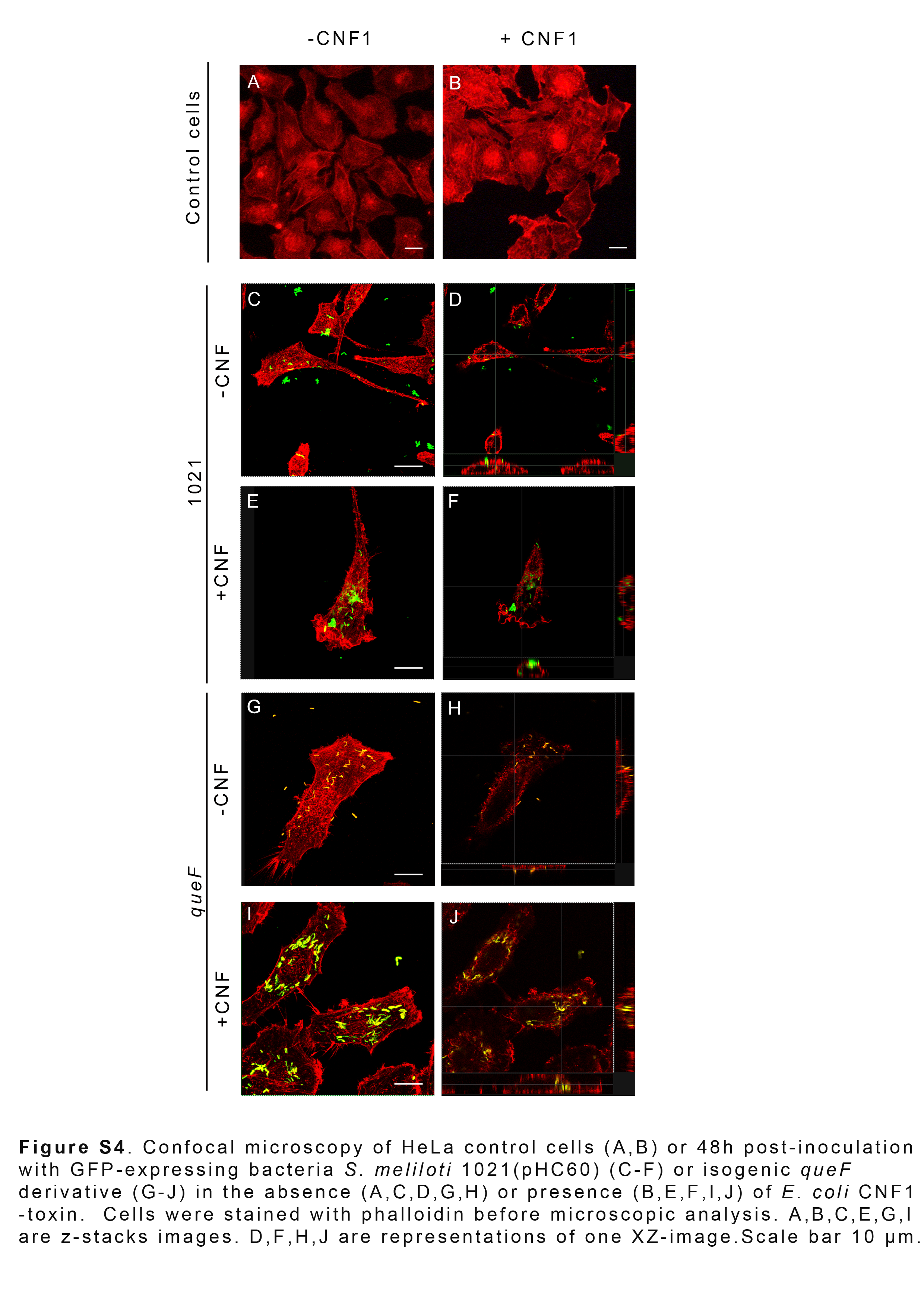

Supplement: Figure S4 — Confocal microscopy of HeLa control cells (A,B) or 48 h post-inoculation with GFP-expressing bacteria S. meliloti 1021(pHC60) (C–F) or isogenic queF derivative (G–J) in the absence (A,C,D,G,H) or presence (B,E,F,I,J) of E. coli CNF1 toxin. Cells were stained with phalloidin before microscopic analysis. A,B,C,E,G,I are z-stacks images. D,F,H,J are representations of one XZ-image. Scale bar 10 µm. (TIF) [file pone.0056043.s004.tif]
