# Supplementary material for: Queuosine Biosynthesis Is Required for Sinorhizobium meliloti-Induced Cytoskeletal Modifications on HeLa Cells and Symbiosis with Medicago truncatula
Source: PLoS One. 2013 Feb 8;8(2):e56043. doi: 10.1371/journal.pone.0056043 (PMC3568095; doi:10.1371/journal.pone.0056043)
Supplement: Figure S3 — Bacterial growth in HeLa cell culture medium (10%FCS). (PPTX) [file pone.0056043.s003.pptx]

## Slide 1
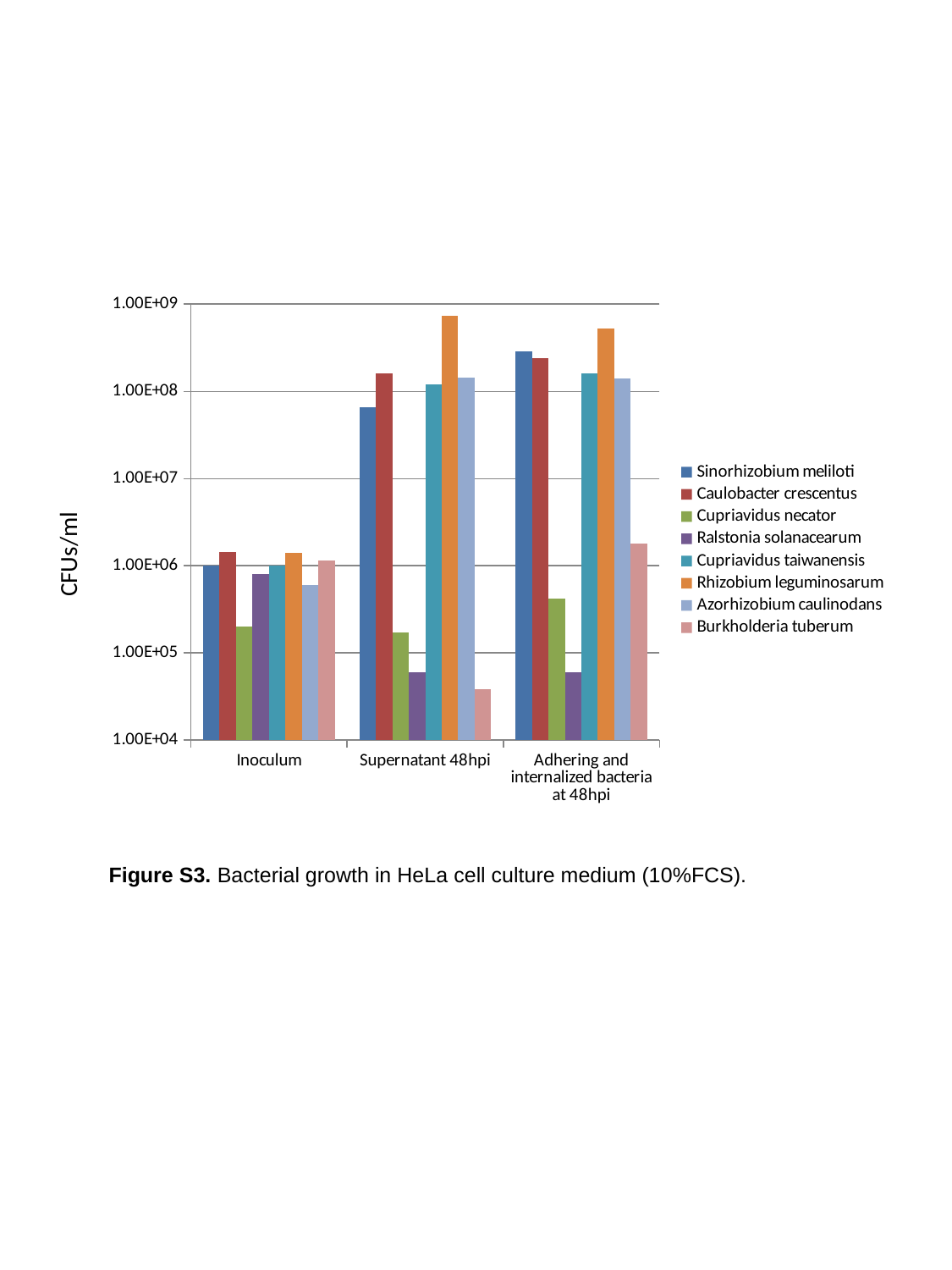

### Chart
| Category | Sinorhizobium meliloti | Caulobacter crescentus | Cupriavidus necator | Ralstonia solanacearum | Cupriavidus taiwanensis | Rhizobium leguminosarum | Azorhizobium caulinodans | Burkholderia tuberum |
|---|---|---|---|---|---|---|---|---|
| Inoculum | 1000000.0 | 1450000.0 | 200000.0 | 800000.0 | 1000000.0 | 1400000.0 | 600000.0 | 1150000.0 |
| Supernatant 48hpi | 66000000.0 | 160000000.0 | 170000.0 | 60000.0 | 120000000.0 | 730000000.0 | 144000000.0 | 38000.0 |
| Adhering and internalized bacteria at 48hpi | 290000000.0 | 240000000.0 | 420000.0 | 60000.0 | 160000000.0 | 530000000.0 | 140000000.0 | 1800000.0 |CFUs/ml
Figure S3. Bacterial growth in HeLa cell culture medium (10%FCS).
