# Supplementary material for: Queuosine Biosynthesis Is Required for Sinorhizobium meliloti-Induced Cytoskeletal Modifications on HeLa Cells and Symbiosis with Medicago truncatula
Source: PLoS One. 2013 Feb 8;8(2):e56043. doi: 10.1371/journal.pone.0056043 (PMC3568095; doi:10.1371/journal.pone.0056043)
Supplement: Figure S5 — Growth of S. meliloti wt 1021 and que/tgt isogenic derivatives in DMEM culture medium with 10%FCS or 0.5% FCS (A), during HeLa cell infection in DMEM medium with 0.5%FCS (B) and in Vincent-succinate medium (C). (PPTX) [file pone.0056043.s005.pptx]

## Slide 1
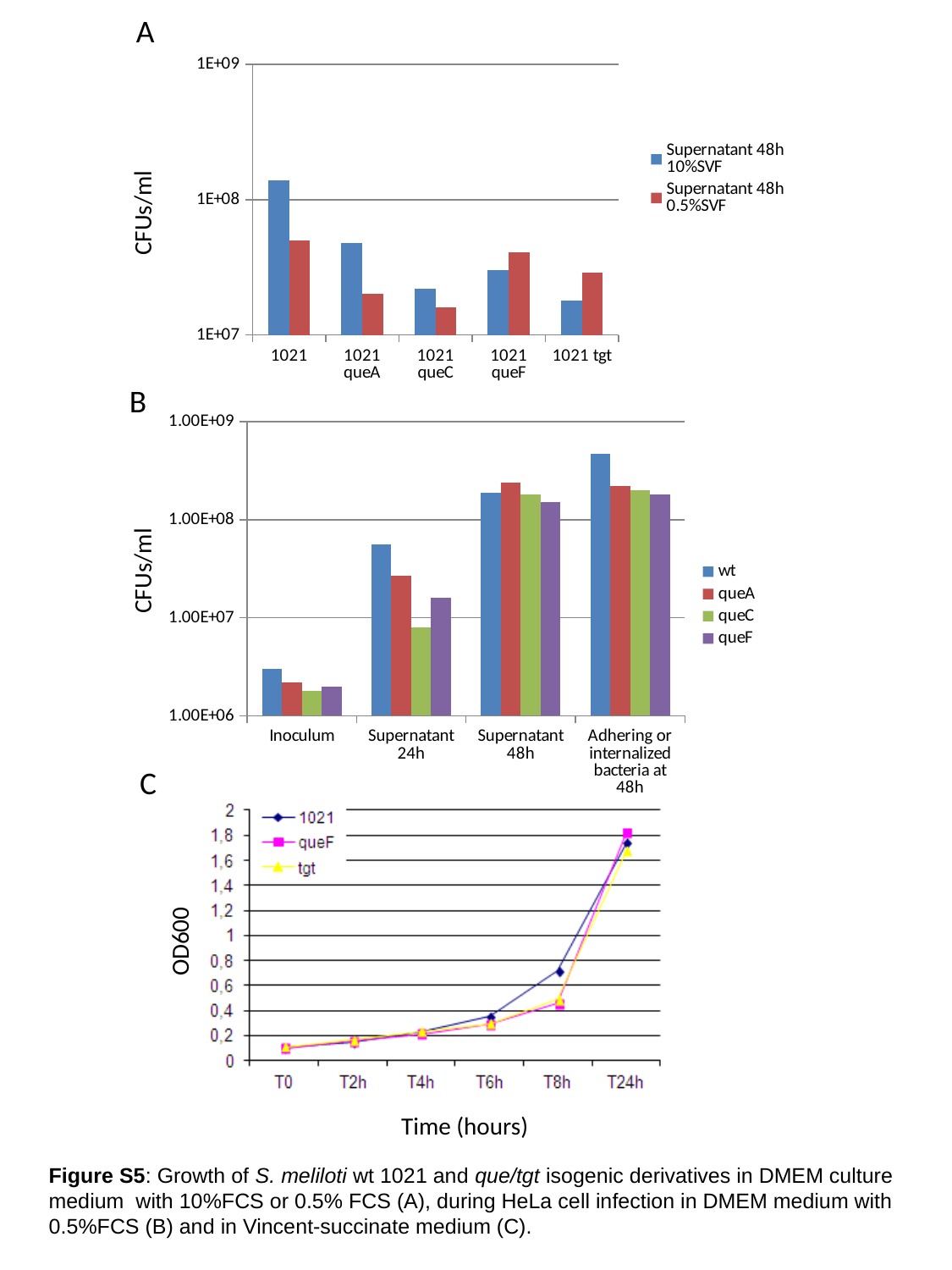

A
### Chart
| Category | Supernatant 48h 10%SVF | Supernatant 48h 0.5%SVF |
|---|---|---|
| 1021 | 140000000.0 | 50000000.0 |
| 1021 queA | 48000000.0 | 20000000.0 |
| 1021 queC | 22000000.0 | 16000000.0 |
| 1021 queF | 30000000.0 | 41000000.0 |
| 1021 tgt | 18000000.0 | 29000000.0 |CFUs/ml
B
### Chart
| Category | wt | queA | queC | queF |
|---|---|---|---|---|
| Inoculum | 3000000.0 | 2200000.0 | 1800000.0 | 2000000.0 |
| Supernatant 24h | 56000000.0 | 27000000.0 | 8000000.0 | 16000000.0 |
| Supernatant 48h | 190000000.0 | 240000000.0 | 180000000.0 | 150000000.0 |
| Adhering or internalized bacteria at 48h | 470000000.0 | 220000000.0 | 200000000.0 | 180000000.0 |CFUs/ml
C
OD600
Time (hours)
Figure S5: Growth of S. meliloti wt 1021 and que/tgt isogenic derivatives in DMEM culture medium with 10%FCS or 0.5% FCS (A), during HeLa cell infection in DMEM medium with 0.5%FCS (B) and in Vincent-succinate medium (C).
