# Supplementary material for: Queuosine Biosynthesis Is Required for Sinorhizobium meliloti-Induced Cytoskeletal Modifications on HeLa Cells and Symbiosis with Medicago truncatula
Source: PLoS One. 2013 Feb 8;8(2):e56043. doi: 10.1371/journal.pone.0056043 (PMC3568095; doi:10.1371/journal.pone.0056043)
Supplement: Figure S6 — Nodulation kinetics of S. meliloti 1021 and queF mutant on M. truncatula seedlings. 20 plants were tested per strain. (PPTX) [file pone.0056043.s006.pptx]

## Slide 1
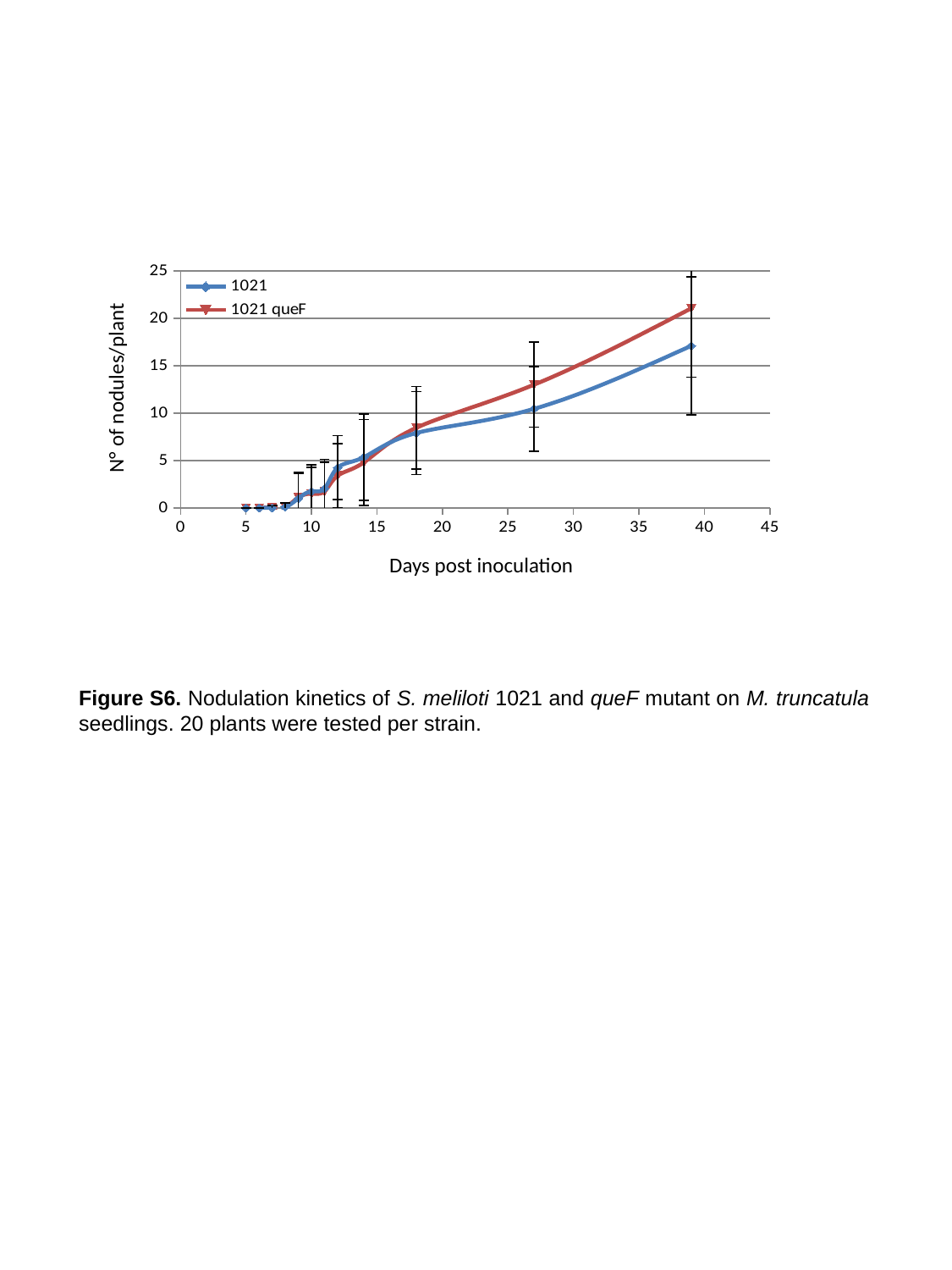

### Chart
| Category | 1021 | 1021 queF |
|---|---|---|N° of nodules/plant
Days post inoculation
Figure S6. Nodulation kinetics of S. meliloti 1021 and queF mutant on M. truncatula seedlings. 20 plants were tested per strain.
