# Supplementary material for: Queuosine Biosynthesis Is Required for Sinorhizobium meliloti-Induced Cytoskeletal Modifications on HeLa Cells and Symbiosis with Medicago truncatula
Source: PLoS One. 2013 Feb 8;8(2):e56043. doi: 10.1371/journal.pone.0056043 (PMC3568095; doi:10.1371/journal.pone.0056043)
Supplement: Table S1 — Effect of S. meliloti mutants on HeLa cells. (DOCX) [file pone.0056043.s007.docx]

**Table S1. Effect of *S. meliloti* mutants on HeLa cells**

| **Mutant  Name** | Mutant  description | **Mutation   localization** | **Cytoskeleton modifications  on HeLa cells** | **Reference** |
| --- | --- | --- | --- | --- |
| A2012 | *nodD1nodD2nodD3* | pSymA | + | [1] |
| GMI3253 | *nodA* | pSymA | + | [2] |
| EK101 | *fixJ* | pSymA | + | [3] |
| VO2119 | *bacA* | pSymB | + | [4] |
| F114 | deletion | pSymB | + | [5] |
| F514 | deletion | pSymB | + | [5] |
| F638 | deletion | pSymB | + | [5] |
| F666 | deletion | pSymB | + | [5] |
| F693 | deletion | pSymB | + | [5] |
| F726 | deletion | pSymB | + | [5] |
| F909 | deletion | pSymB | + | [5] |
| G373 | deletion | pSymB | + | [5] |
| G462 | deletion | pSymB | - | [5] |
| G470 | deletion | pSymB | + | [5] |
| G471 | deletion | pSymB | + | [5] |
| G472 | deletion | pSymB | + | [5] |
| G536 | deletion | pSymB | - | [5] |
| Rm5378 | deletion | pSymB | - | [5] |
| Rm5416 | deletion | pSymB | - | [5] |
| GMI11660 | Δ(*exsH*-SMb20972) | pSymB | - | This work |
| GMI11661 | Δ (SMb21581-*exsA*) | pSymB | - | This work |
| GMI11662 | Δ (*thtR*-SMb21591) | pSymB | + | This work |
| GMI11663 | *exoI* | pSymB | + | This work |
| GMI11664 | *exoT* | pSymB | + | This work |
| GMI11665 | *exoW* | pSymB | + | This work |
| Rm7210 | *exoY* | pSymB | + | [6] |
| GMI11666 | *exsH* | pSymB | + | This work |
| GMI11656 | *queC (exsB)* | pSymB | - | This work |
| GMI11655 | *queA* | Chromosome | - | This work |
| GMI11546 | *queF* | Chromosome | - | This work |
| GMI11658 | SMc02721 | Chromosome | + | This work |
| GMI11659 | SMc02722 | Chromosome | + | This work |
| GMI11657 | *tgt* | Chromosome | - | This work |
| SMGC5 | *lpsB* | Chromosome | + | [7] |
| GMI11667 | SMc04270 | Chromosome | + | [8] |
| EK257 | *typA* | Chromosome | + | [8] |
| GMI11668 | *fecR* (SMc4204) | Chromosome | + | This work |
| GMI11567 | *clr* | Chromosome | + | [9] |

**References**

1. Honma MA, Asomaning M, Ausubel FM (1990) RHIZOBIUM-MELILOTI NODD GENES MEDIATE HOST-SPECIFIC ACTIVATION OF NODABC. Journal of Bacteriology 172: 901-911.

2. Debelle F, Rosenberg C, Vasse J, Maillet F, Martinez E, et al. (1986) ASSIGNMENT OF SYMBIOTIC DEVELOPMENTAL PHENOTYPES TO COMMON AND SPECIFIC NODULATION (NOD) GENETIC-LOCI OF RHIZOBIUM-MELILOTI. Journal of Bacteriology 168: 1075-1086.

3. Bobik C, Meilhoc E, Batut J (2006) FixJ: a major regulator of the oxygen limitation response and late symbiotic functions of Sinorhizobium meliloti. Journal of Bacteriology 188: 4890-4902.

4. Oke V, Long SR (1999) Bacteroid formation in the Rhizobium-legume symbiosis. Current Opinion in Microbiology 2: 641-646.

5. Charles TC, Finan TM (1991) ANALYSIS OF A 1600-KILOBASE RHIZOBIUM-MELILOTI MEGAPLASMID USING DEFINED DELETIONS GENERATED INVIVO. Genetics 127: 5-20.

6. Leigh JA, Signer ER, Walker GC (1985) EXOPOLYSACCHARIDE-DEFICIENT MUTANTS OF RHIZOBIUM-MELILOTI THAT FORM INEFFECTIVE NODULES. Proceedings of the National Academy of Sciences of the United States of America 82: 6231-6235.

7. Campbell GRO, Reuhs BL, Walker GC (2002) Chronic intracellular infection of alfalfa nodules by Sinorhizobium meliloti requires correct lipopolysaccharide core. Proceedings of the National Academy of Sciences of the United States of America 99: 3938-3943.

8. Becker A, Berges H, Krol E, Bruand C, Ruberg S, et al. (2004) Global changes in gene expression in Sinorhizobium meliloti 1021 under microoxic and symbiotic conditions. Molecular Plant-Microbe Interactions 17: 292-303.

9. Tian ZX, Mao XJ, Su W, Li J, Becker A, et al. (2006) Exogenous cAMP upregulates the expression of glnII and glnK-amtB genes in Sinorhizobium meliloti 1021. Chinese Science Bulletin 51: 1982-1985.
