# Supplementary material for: Queuosine Biosynthesis Is Required for Sinorhizobium meliloti-Induced Cytoskeletal Modifications on HeLa Cells and Symbiosis with Medicago truncatula
Source: PLoS One. 2013 Feb 8;8(2):e56043. doi: 10.1371/journal.pone.0056043 (PMC3568095; doi:10.1371/journal.pone.0056043)
Supplement: Table S2 — Oligonucleotides used for the construction of strains and plasmids. (DOC) [file pone.0056043.s008.doc]

**Table S2.** Oligonucleotides used for the construction of strains and plasmids

| **Name** | **Sequence** | **Name** | **Sequence** | **Constructions** |
| --- | --- | --- | --- | --- |
| ODC183 | **CGGGATCC**GGCTGAACAGGAGCTTTCC | ODC184 | **GCTCTAGA**GGCAATCGGGATAGATGAAG | 1021 *queC* mutant |
| ODC217 | **CGGGATCC**ATCGGGACTTTCACAACTGG | ODC218 | **GCTCTAG**AGCCACTTCGGAGAAAGCAG | 1021 *queF* mutant |
| ODC219 | **CGGGATCC**ACCGGGGTGAGGATATAAGC | ODC220 | **GCTCTAGA**GCGCATAGACGGTCTGGTAG | 1021 *queA* mutant |
| ODC227 | **CGGGATCC**AGGCGATGTATCTCGACCAG | ODC228 | **GCTCTAGA**TCGAGCTGCATCTGAATGTC | 1021 *tgt* mutant |
| ODC233 | **CGGGATCC**TAAATCGGATGGCTTCAAGG | ODC234 | **GCTCTAGA**GGAGTCGTTACGGACATCGT | 1021 SMc02721 mutant |
| ODC235 | **CGGGATCC**GGTGACGAAATTCTGCTTCC | ODC236 | **GCTCTAGA**GCCAGGCTTTCTTGAGGTC | 1021 SMc02722 mutant |
|  |  |  |  |  |

Primer extensions for restriction sites are indicated in bold.
